# Survival of Bonded Space Maintainers: A Systematic Review

**DOI:** 10.5005/jp-journals-10005-1554

**Published:** 2018-10-01

**Authors:** Shantanu S Deshpande, Vikas D Bendgude, Vivian V Kokkali

**Affiliations:** 1 Postgraduate Student, Department. of Pedodontics, Dr. D.Y Patil Dental College, Pimpri, Pune, Maharashtra, India; 2 Professor and HOD, Department. of Pedodontics, Dr. D.Y Patil Dental College, Pimpri, Pune, Maharashtra, India; 3 Senior Lecturer, Department. of Pedodontics, Dr. D.Y Patil Dental College, Pimpri, Pune, Maharashtra, India

**Keywords:** Bonded space maintainers, Longevity, Survival rate.

## Abstract

**Aim:**

This systematic review was aimed at evaluating the average survival time and the various factors which determine the longevity of bonded space maintainers.

**Background:**

Although a meta-analysis could not be performed from the available literature, this review emphasizes the various factors contributing to the success of bonded space maintainers and its relevance during the planning of bonded space maintainers.

**Review results:**

The study selection criteria included in-vivo randomized and non-randomized clinical trials performed which was published in English. The databases searched were Pubmed, EBSCOhost and Google scholar, wherein the articles published from 1st January 1995 to 31st December 2015 were selected in the review.

**Conclusion:**

From the existing data, it can be concluded that the average survival period of bonded space maintainers is 11.2 months. However, there is a necessity for additional clinical trials with strict protocols to better the level of evidence.

**Clinical significance:**

From the various articles included in the review, the longevity of bonded space maintainers was found to be comparable to the banded space maintainers. Hence, the bonded space maintainers can be a suitable alternative to the banded space maintainers in pediatric dentistry.

**How to cite this article:** Deshpande SS, Bendgude VD, Kokkali VV. Survival of Bonded Space Maintainers: A Systematic Review Int J Clin Pediatr Dent. 2018;11(5):440-445.

## BACKGROUND

Space maintenance is an indispensable part of Pediatric Dentistry and is the primary preventive orthodontic care that is provided to avoid future dental anomalies. Primary teeth play a critical role in the growth and development of children. Although they are used for mastication, speech, growth of the jaws, esthetics, and guidance of normal function; primary teeth most importantly serve as a natural space maintainer for their permanent successors.^1^

Pre-mature loss of primary teeth, especially the molars could eventually lead to mesial drifting of the posterior teeth, crowding in the dental arches, changes in the arch circumference, which may eventually result in insufficient space for the eruption ofthe permanent teeth.^2-4^ The use of space maintainers is the recommended treatment for the protection of dental arch relations.

Space maintainers can be broadly classified as removable or fixed space maintainers.^1^ Removable space maintainers are usually functional and are easy to clean which can help in maintaining good oral hygiene. However, the success of these removable appliances heavily weighs on the patient compliance and considering that the patients are mostly children, compliance becomes a problem. Moreover, there is always a chance for these appliances of being fractured or misplaced.^5^ On the other hand, fixed space maintainers reduce the need for patient compliance, require minimal care, and are relatively comfortable and acceptable to the patient.

The conventionally fixed space maintainersare mostly banded.^6^ It requires less chair-side time, easy to fabricate, adapts easily to the changing dentition and is extremely economical. Although the banded applianceis being used successfully, they do have certain disadvantages like:^4^

- Two visits are required, therefore cannot be planned in patients under general anesthesia.
- May lead to tipping and rotation of the abutment teeth.
- Occasionally, requires some preparation in the abutment teeth.
- Requires laboratory procedures.
- Dislodgment of the band due to loss of the luting cement
- Impingement of the soft tissue because of slipping of the loop gingivally as it is a cantilever type of an appliance.
- Plaque accumulation at the band-tooth interface could lead to incipient carious lesions and gingival inflammation.
- There also have been cases of metal allergy.^7-9^

These disadvantages have led clinicians to develop a more viable alternative to the traditional space maintainers. Swaine et al.^10^ was the first to use bonded space maintain-ers and reporteda 70% success rate. Over the past few decades, adhesive technology is being harnessed to replace the conventional space maintainer in the form of direct bonded space maintainers (Ribbond^®^), fiber-reinforced composite space maintainers (Super Splint^®^), and prefabricated space maintainers (Splint-In^®^). Also, orthodontic stainless steel wires have been used in the form of a loop and directly bonded to the tooth.

The bonded space maintainers have certain advantages over the conventional banded space maintainers. They can be delivered to the patient in a single appointment, eliminating the need for laboratory procedures. Moreover, it also reduces the chances of plaque accumulation which helps maintain the health of hard and soft tissues in the oral cavity. The existing literature on bonded space maintainer, evaluates the following parameters, namely, the survival period, gingival and periodontal health, condition of the abutment tooth and the time required for fabrication of the appliance. Survival of the space maintainer until the eruption of the succedaneous tooth is the most important factor in determining the success of the bonded appliance as it measures the primary function of space maintenance.

Hitherto, no systematic reviews have been performed on the survival time of bonded space maintainers. Hence, this systematic review was aimed at evaluating, the average survival time andthe factors which determine the longevity of bonded space maintainers.

## DATA SOURCES

This systematic review was performed in accordance with the Preferred Reporting Items for Systematic Reviews and Meta-Analyses (PRISMA) guidelines. A systematic computerized search was performed on three electronic databases: PubMed, EBSCOhost and Google Scholar. The various keywords used to search articles in the PubMed and EBSCOhost databases were as follows:

- “Space maintainers and survival rate.”
- “Space maintainers and longevity.”
- “Space maintainers and maxillary arch.”
- “Space maintainers and mandibular arch.”
- “Fixed space maintainers and survival rate.”
- “Fixed space maintainers and longevity.”
- “Bonded space maintainers and survival rate.”
- “Bonded Space maintainers and longevity.”

Hand searches were undertaken to find additional relevant published material that might have been missed in electronic searches. The articles published from 1st January 1995 to 31st December 2015 were included in the study.

In the first step of the screening process, titles and abstracts were used to identify full articles concerning the survival times of bonded space maintainers used in the pediatric populations.

In the second step of the screening process, the duplicates from the respective searches were removed, and one single article was selected. In the third step, these articles were subjected to the inclusion and exclusion criteria of the review.

### Inclusion Criteria

- Articles in English or those having a detailed summary in English.
- Studies published between 1st January 1995 and 31st December 2015.
- Studies that provided information for age groups < 18 from either sex.
- Well defined information on the survival rate of bonded space maintainers in maxillary and man-dibular arches.

### Exclusion Criteria

- Review
- Case reports
- Abstracts
- Letters to editors
- Editorials
- In-vitro studies.

All the studies identified by applying the inclusion and exclusion criteria underwent assessment for data extraction by a single reviewer. The data were extracted using specifically designed data extraction forms. For each included study, the qualitative and quantitative information was extracted, including year of publication, experimental and control treatments, numbers and ages of patients, treatment and follow-up durations, author’s conclusions and all the information needed for methodo-logic quality evaluation.

## REVIEW RESULTS

**Fig. 1:**
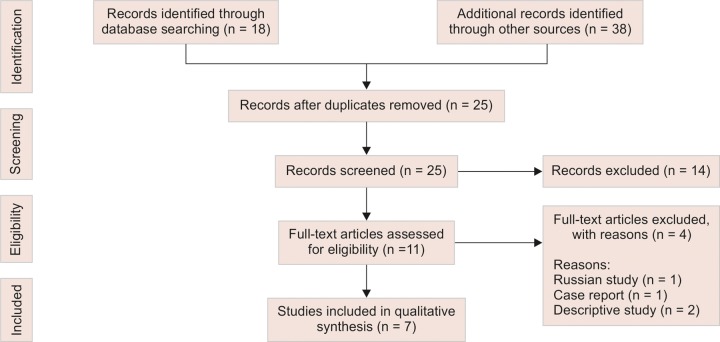
Prisma flow diagram

The database search showed 18 articles on Pubmed, 21 articles on EBSCOhost and 13 articles on Google Scholar. Four articles were added after hand searches of the bibliographies of the selected articles. By using the PRISMA flow diagram (Fig. 1), an overview of the article selection process can be illustrated. After exclusion of the duplicate articles 25 articles were finally selected for the study.

In the first step of the screening process, further 14 articles were excluded because they were determined to be irrelevant based on titles and abstracts. In the second step of the screening process, further four articles were excluded because they did not meet the inclusion and exclusion criteria (Table 1). Thus, the selection process resulted in seven full-text articles (Table 2).

## DISCUSSION

In spite of many studies investigating the survival rate of bonded space maintainers, only seven studies were considered appropriate for inclusion in this systematic review. It was not possible to formulate a quantitative conclusion since the study designs were very different from each other.

**Table 1:** **Table 1:** Excluded articles with reasons

*Sr*				*Year of*			
*No*		*Study*		*publication*		*Reason for exclusion*	
1		Yilmaz et al.		1999		Only abstract in English	
2		Negi KS		2010		Descriptive study	
3		Bhasin et al.		2011		Case report	
4		Yeluri et al.		2012		Descriptive study	

**Table 2:** **Table 2:** Selected articles

*Sr no*		*Study*		*Year of publication*	
1		Simsek et al.		2004	
2		Kirzioglu et al.		2004	
3		Subramaniam P et al.		2008	
4		Emine Sen Tunc et al.		2012	
5		Setia et al.		2014	
6		Garg et al.		2014	
7		Serkan Gulec et al.		2014	

Relative to the study designs included in this review, two were non-randomized clinical trials,^4,11^ four studies were randomized clinical trials,^12-15^ and one was an experimental study^16^ evaluating the survival rate of bonded space maintainers. The summary of all the included articles has been described in Table 3. The advantage of a selection of randomized clinical trials over cohort and non-randomized trails is the random allocation and avoidance of bias.

There are different criteria to evaluate the success of bonded space maintainers. These include the longevity of space maintainers, gingival health, plaque accumulation, the condition of the abutment tooth and the ease to fabricate the appliance. The most important being their longevity and their ability to maintain space which is their primary function. From the selected studies, the patients were being followed-up from 9 to 18 months. On an average, the survival time of bonded space maintainers was 11.2 months depending upon the type of space maintainer placed and the age of the patient at the time of placement. However, the maximum period a bonded space maintainer that sustained in the oral cavity was 15.3 months.^4^ This bonded appliance was fabricated using orthodontic stainless steel wire, in which the loop was bonded to permanent molars using a Single Bond^^®^^ system and Tetric Flow^^®^^ composite resin. The study was carried out in children with an average age of 7.3 years which is ideal because the chronological age of eruption of premolars is 10 to 12 years.

The parameters that determine the longevity include the type of isolation technique used, arch in which space maintainers were given, the bonding systems used, composite kind of resin used and the abutment tooth (primary or permanent tooth) being bonded.

The various methods of isolation like cotton rolls and saliva ejectors have been used,^16^ but the rubber dam provides optimum isolation. The rubber dam when used makes moisture contamination negligible.^12,15^ This, in turn, enhances the bonding of the space maintainers to the abutment tooth. Hence, the use of rubber dam should be encouraged during placement of bonded space maintainers. Similarly, moisture control is better in the maxillary arch as compared to the mandibular arch. Therefore, it has been observed in few studies; survival time of bonded space maintainers in the maxillary arch was better as compared to the opposing lower arch.^4,11^

**Table 3:** **Table 3:** Summary of the included studies in terms of study design and results

*Study ID*		*Author*		*Participants*		*Intervention*		*Comparison*		*Outcomes*		*Study design*		*Follow up*		*Main findings*	
1		Simsek et al.^4^		74 space maintainers in 51 children (19 girls and 32 boys) Avg age = 7.3 years, maintainers in 51 children (19 girls and 32 boys) Avg age = 7.3 years.		Fixed space maintainer bonded with a composite resin		None		Space Maintenance		Nonrandomized clinical trial		Till the permanent tooth erupts		Out of 64 space maintainers, 34 space maintainers were placed in mandible and 30 in maxilla with a mean survival time of 12 to 18 months, (average time being 15.3 months). This study showed a moderate risk of bias.	
2		Kirzioglu et al.^11^		40 space maintainers in 29 children (14 girls, 15 boys) Aged 7-14 years Avg age = 10 years and 1 month		Fixed space maintainer bonded with Sprint- In		None		Space maintenance		Nonrandomized clinical trial		Till the permanent tooth erupts		Out of the 40 space maintainers, 24 space maintainers were placed in mandible and 16 in maxilla with a mean survival time of 5.7 months. This study has a moderate risk of bias	
3		Subramaniam et al.^12^		60 space maintainers in 30 children (7 girls and 23 boys) aged 6-8 years		Fibre reinforced the composite resin		Band and loop space maintainer		Longevity of space maintainers		Randomized clinical trial		12 months		Out of the 60 space maintainers placed on 26 survived at the end of 12 months. This study has a moderate risk of bias.	
4		Tunc et al.^13^		30 space maintainers in 30 children (15 girls and 15 boys) aged 4-10 years.		Fibre reinforced the composite resin		Band and loop space maintainers and directly bonded space maintainers.		Longevity of space maintainers		Randomized clinical trial		12 months		The mean survival time of fixed space maintainers was 9.03 months with band and loop for 11.2 months followed by 9.2 months for directly bonded space maintainers and 6.7 months for fiber reinforced composite resin. This study has a moderate risk of bias.	
5		Setia et al.^14^		60 space maintainers in 60 children aged 4-9 years		Ribbond and Super Splint		Band and loop space maintainers and prefabricated band with custommade loops		Longevity of space maintainers		Randomized clinical trial		9 months		The success rate of the prefabricated band with custom-made loops at the end of 9 months was 84.6%, whereas super splint was 33.33%, ribbond is 45.4 %, and band and loop is 73.3%. This study has a high risk of bias.	
6		Garg et al.^15^		60 space maintainers in 30 children aged 5-8 years		Fibre reinforced the composite resin		Band and loop space maintainers		Longevity of space maintainers		Randomized clinical trial		18 months		Out of 42 space maintainers analyzed at 18 months, fiber reinforced composite resin showed a success rate of 63.3% and band and loop showed a success rate of 36.7%. This study has a moderate risk of bias.	
7		Gulec et al^16^		41 space maintainers in 27 children (11 girls and 16 boys) aged 6 to 12 years.		EZ space maintainer		None		Survival of EZ space maintainers		Experimental study		Till the failure of the appliance		The mean survival time of EZ space maintainer is 220 days. This study has a moderate risk of bias.	

The resin to tooth bond strength also plays a vital role in determining the longevity of the bonded space maintainer. The bond strength of primary tooth enamel is considerably lower than the permanent tooth enamel. This can be attributed to the presence of prismless zones in the enamel of primary teeth, that tend to have an adverse effect on the bond strength thereby, affecting resin retention.^17^ The bond strength of the resin can be positively modified by grinding the outer enamel layer and increasing the etching time.^18-20^ The bonding, however, is better in permanent teeth than in primary teeth. However, in cases of permanent first molars in the mandible, Artun stated that occlusal trauma might be more of a problem in cases of newly erupted teeth where area available for bonding is inadequate. The main reasons for failure in the enamel-composite bond are the improper surface preparation, moisture contamination, and disturbances during the adhesive setting process.^21^ In the majority of the studies, a fifth-generation bonding agent was used along with a flow composite resin. Fifth generation bonding system has better bonding abilities and fewer steps required. Flow composite resin, which is low in filler particles have low viscosity and better flow leading to the emergence of lower air bubbles during application. This helps in better penetration in the tooth surfacewhich aids in bonding. Adequate bond strength was also achieved when bonded with the packable form of composites (Transbond^®^).^16^

The studies in which the space maintainers were fabricated with an orthodontic stainless steel wire, the survival time was higher (15.3 months) as compared to the fiber-reinforced composite resin (FRCR) material (9-12 months) This can be attributed to the fact that the orthodontic stainless steel wire was fabricated following the contours of the ridge and hence, the tooth of the opposing arch did not impinge on the loop during occlusion. The FRCRs and the NiTi wires were bonded directly on to the abutment teeth in a horizontal fashion which did not follow the contours of the ridge. This design, therefore, exposed the loop to the occlusal forces which was an important reason for the fracture of the fiber frame. With longer time-interval, there is a possibility of supra-eruption of the opposing tooth which eventually impinges on the fiber frame. This also could result in increased concentration of mechanical stresses on the fiber frame and its subsequent fracture.^12^ Another type of failure observed was debonding at the composite-fiber interface. This type of failure occurs due to overzealous finishing that caused the excess removal of the resin overlying the fiber.^12^

The factors like the bonding systems, the type of abutment tooth used, the material used for fabrication and the design of the loop, if used appropriately will enhance the survival rate of bonded space maintainer. The meticulous oral hygiene maintenance and periodic recall are also important to achievea higher success rate.

The type of studies included in the systematic review consisted of randomized and non-randomized clinical trials only. Hence, the literature available in other types of studies like the case study, case reports, case series, *in-vitro* studies was not taken into consideration while formulating the results of this review. This review did not include studies published post 31st December 2015. Such studies also formed a part of the exclusion criteria. Since the language of the review was English, articles published in any other languages were excluded from the study. This may also result in prevailing us about some literature on the topic.

Improper selection criteria and/or sample size calculation and/or lack of randomization while performing the intervention in most of the included studies resulted in moderate to high risk of bias. Hence, the available literature fails to have a high level of evidence when it comes to treatment selection. To improve the level of evidence, it is suggested that future studies should include control groups, define strict patient inclusion and exclusion criteria and conduct the radiographic evaluation pre- and post-operatively. The intended clinical findings should be clearly stated and appropriate randomization protocols also need to be included to avoid the risk of bias.

## CONCLUSION

The existing literature provides evidence-based knowledge about the longevity of bonded space maintainers. The existing data support the use of rubber dam for good isolation, also, when bonding to primary dentition, surface modifications of the enamel and increased etch time are mandatory. The fifth-generation bonding agents and flowable type of composite resins are recommended for enhancing bond strength. The best design of the bonded appliance would be the one in which the components are not in the plane of occlusion, hence, an orthodontic stainless steel wire following the contours of the arch bonded to an abutment tooth would be ideal. Appropriate design and fabrication, meticulous oral hygiene maintenance, and regular follow-ups would certainly make bonded space maintainers a viable alternative to the conventional banded appliances.

## CLINICAL SIGNIFICANCE

In accordance to the articles included in the systematic review, it can be taken into consideration that bonded space maintainers serve as a viable alternative to the banded space maintainers in pediatric dentistry.

## References

[1] Bijoor RR, Kohli K (2005). Contemporary space maintenance for the pediatric patient. N Y State Dent J.

[2] Baroni C, Franchini A, Rimondini L (1994). Survival of different types of space maintainers. Pediatr Dent.

[3] Yilmaz Y, Kocogullari ME, Belduz N (2006). Fixed space main-tainers combined with open-face stainless steel crowns. J Contemp Dent Pract.

[4] Simsek S, Yilmaz Y, Gurbuz T (2004). Clinical evaluation of simple fixed space maintainers bonded with flowable com-posite resin. J Dent Child.

[5] Kargul B, Caglar E, Kabalay U (2003). Glass fiber-reinforced composite resin space maintainer: Case reports. J Dent Child.

[6] Mathewson RJ, Primosch RE (1995). Fundamentals of Pediatric Dentistry..

[7] Nayak UA, Loius J, Sajeev R, Peter J (2004). Band and loop space maintainer-made easy. J Indian Soc Pedod Prev Dent.

[8] Qudeimat MA, Fayle SA (1998). The longevity of space maintainers: A retrospective study. Pediatr Dent.

[9] Caroll TP (1982). Prevention of gingival submergence of fixed unilateral space maintainers. J Dent Child.

[10] Swaine TJ, Wright GZ (1975). Direct bonding applied to space maintenance. ASDC journal of dentistry for children..

[11] Kirzioglu Z, Erturk O, Semra M (2004). Success of reinforced fibre material space maintainers. Journal of dentistry for chil-dren..

[12] Subramaniam P, Babu GK, Sunny R (2008). Glass fibre-reinforced composite resin as a space maintainer: A clinical study. Journal of Indian Society Of Pedodontics and Preventive Dentistry..

[13] Tunc Es, Bayrak S, Tuloglu N, Egilmez T, Isci D (2012). Evaluation of survival of 3 different fixed space maintainers. Pediatric Dentistry..

[14] Setia V, Pandit IK, Srivastava N, Gugnani N, Gupta M (2014). Banded vs Bonded Space Maintainers: Finding Better Way Out. International journal of clinical pediatric dentistry..

[15] Garg A, Samadi F, Jaiswal JN, Saha S (2014). ’Metal to resin’: A comparative evaluation of conventional band and loop space maintainer with the fiber reinforced composite resin space maintainer in children. J Indian! Soc Pedod Prev Dent.

[16] Gulec S, Dogan MC, Seydaoglu G (2014). Clinical evaluation of a new bonded space maintainer. Journal of clinical orthodontics: JCO.

[17] Bhasin AS (2011). Simplified Bonded Space Maintainer- A Case Report. Journal Of the Indian Dental Association.

[18] Swartz ML, Philips RW, Clark HE (1984). Long-term F release from glass ionomer cements. J Dent Res.

[19] Millett DT, McCabe JF, Bennett TG, Carter NE, Gordon PH (1995). The effect of sandblasting on the retention of first molar orthodotic bands cemented with glass ionomer cement. Br J Orthod.

[20] Swaine TJ, Wright GZ (1976). Direct bonding applied to space maintenance. J Dent Child.

[21] Zachrisson BU (1977). Clinical experience with direct bonding in orthodontics. Am J Orthod.

